# Radiographic knee osteoarthritis is associated with higher lumbar spine bone mineral density but not reduced vertebral fracture prevalence: a retrospective cross-sectional study in older Asian women

**DOI:** 10.1186/s12891-026-09751-8

**Published:** 2026-03-30

**Authors:** Yi-Chieh Huang, Sheng-Wen Kao, Yi-Chou Chen, Ming-Te Cheng

**Affiliations:** 1 Sinwu Branch, Taoyuan General Hospital, Ministry of Health and Welfare, Taoyuan City, Taiwan; 2 Department of Orthopaedic Surgery, Taoyuan General Hospital, Ministry of Health and Welfare, Taoyuan City, Taiwan; 3 Graduate Institute of Medical Sciences, National Defense Medical Center, Taipei City, Taiwan; 4 Department of Biomedical Engineering, Chung Yuan Christian University, Taoyuan City, Taiwan; 5 Department of Medical Science, National Tsing Hua University, Hsinchu, Taiwan

**Keywords:** knee osteoarthritis, bone mineral density, dual-energy X-ray absorptiometry, vertebral fracture

## Abstract

**Background:**

The relationship between knee osteoarthritis (OA), bone mineral density (BMD), and vertebral fractures remains controversial. Degenerative changes associated with OA may artifactually elevate lumbar spine BMD measured by dual-energy X-ray absorptiometry (DXA), potentially obscuring true skeletal fragility. We examined the association of radiographic knee OA with site-specific BMD and vertebral fracture prevalence in older Asian women.

**Methods:**

This retrospective cross-sectional study included 2,036 postmenopausal women who underwent knee radiography, lumbar spine and hip DXA, and lateral thoracolumbar radiography (T4–L4) during health examinations at a single center. Radiographic knee OA was defined as Kellgren–Lawrence grade ≥ 2. Lumbar spine and hip BMD T-scores were recorded. Vertebral fractures were assessed using a semi-quantitative radiographic approach. Logistic regression analyses were performed to evaluate associations between knee OA and prevalent vertebral fractures, adjusting for age and body mass index (BMI).

**Results:**

Radiographic knee OA was present in 27.0% of participants. Women with OA were older and had higher BMI. Lumbar spine T-scores were significantly higher in women with OA, whereas hip T-scores were not independently associated with OA after adjustment. Vertebral fractures were more prevalent in women with OA in crude analyses (41.5% vs. 30.7%), but this association was fully attenuated after adjustment for age and BMI (adjusted OR 0.95, 95% CI 0.76–1.20).

**Conclusions:**

In this cross-sectional sample of older Asian women, radiographic knee OA was associated with higher lumbar spine BMD but not with lower vertebral fracture prevalence after accounting for age and BMI. Elevated spine BMD in OA may reflect degenerative artifact rather than enhanced skeletal strength. In patients with knee OA, hip BMD and clinical fracture history may provide more reliable indicators of skeletal fragility than lumbar spine BMD alone.

**Supplementary Information:**

The online version contains supplementary material available at 10.1186/s12891-026-09751-8.

## Introduction

Population aging has led to a rapid increase in chronic musculoskeletal disorders worldwide, particularly knee osteoarthritis (OA) and osteoporosis. Knee OA is a major cause of pain, disability, and reduced mobility in older adults, whereas osteoporotic vertebral fractures are associated with substantial morbidity, impaired quality of life, and increased mortality [1, 2]. As life expectancy increases, the coexistence of OA and osteoporosis in older women has become increasingly common in clinical practice [3–5]. Clarifying the relationship between these conditions is therefore of considerable epidemiologic and clinical importance.

For several decades, studies have reported a paradoxical association between OA and bone mineral density (BMD). Individuals with radiographic OA frequently demonstrate higher BMD at axial skeletal sites compared with those without OA [6–9]. This observation has contributed to the perception that OA may be protective against osteoporosis. However, dual-energy X-ray absorptiometry (DXA) measures areal bone density and may be influenced by degenerative changes, particularly in the lumbar spine. Osteophyte formation, facet joint hypertrophy, and endplate sclerosis can artifactually elevate measured lumbar spine BMD [10, 11], potentially obscuring true skeletal fragility.

Importantly, elevated axial BMD in OA populations does not necessarily translate into lower fracture prevalence. Several epidemiologic studies have reported that individuals with knee OA may exhibit similar or even higher rates of vertebral fractures despite normal or elevated spine BMD [12–16]. These findings suggest that the apparent OA–BMD relationship may reflect measurement artifact or shared confounding factors rather than enhanced skeletal strength, with emerging genetic evidence further supporting a complex and potentially bidirectional relationship between OA and BMD [12, 16–19]. Moreover, the association appears to be site-specific. While lumbar spine BMD may be artificially elevated, hip BMD—less affected by degenerative artifact—has shown inconsistent associations with knee OA [12, 16–19]

Despite extensive investigation, important gaps in knowledge remain. First, many prior studies have examined OA and BMD without simultaneously evaluating clinically relevant fracture outcomes. Second, available data are derived predominantly from Western populations, whereas older Asian women exhibit distinct skeletal characteristics, a high prevalence of vertebral fractures, and potentially different OA phenotypes. Third, few studies have evaluated the OA–fracture relationship while explicitly accounting for major confounders such as age and body composition within the same analytic framework.

Given these uncertainties, further clarification is warranted. The present study aimed to examine the association between radiographic knee OA, site-specific BMD, and vertebral fracture prevalence in a large sample of older Asian women. Using a cross-sectional design, we sought to determine whether elevated lumbar spine BMD in women with knee OA corresponds to lower vertebral fracture prevalence after adjustment for age and body mass index. By integrating radiographic OA assessment, DXA-derived BMD at multiple skeletal sites, and vertebral fracture evaluation within a single cohort, this study provides epidemiologic insight into skeletal health in women with knee OA.

## Materials and methods

### Study design and setting

This was a retrospective cross-sectional study conducted at Taoyuan General Hospital, Taiwan. The study included community-dwelling postmenopausal women who underwent standardized health examinations between January 2021 and December 2024. Participants were self-referred for comprehensive health screening rather than symptom-based referral. The health examination package included anthropometric measurements, dual-energy X-ray absorptiometry (DXA), standing knee radiography, and lateral thoracolumbar radiography (T4–L4).

The study protocol was approved by the Institutional Review Board of Taoyuan General Hospital (approval no. TYGH114057). The requirement for informed consent was waived due to the retrospective design and use of de-identified data.

### Participants

Women aged ≥ 50 years who underwent standing knee radiography, lumbar spine and hip DXA, and lateral thoracolumbar radiography (T4–L4) during the same examination visit were eligible. Exclusion criteria were:

- Prior bilateral total knee arthroplasty
- Inadequate image quality
- Missing key demographic or imaging data

**Fig. 1:**
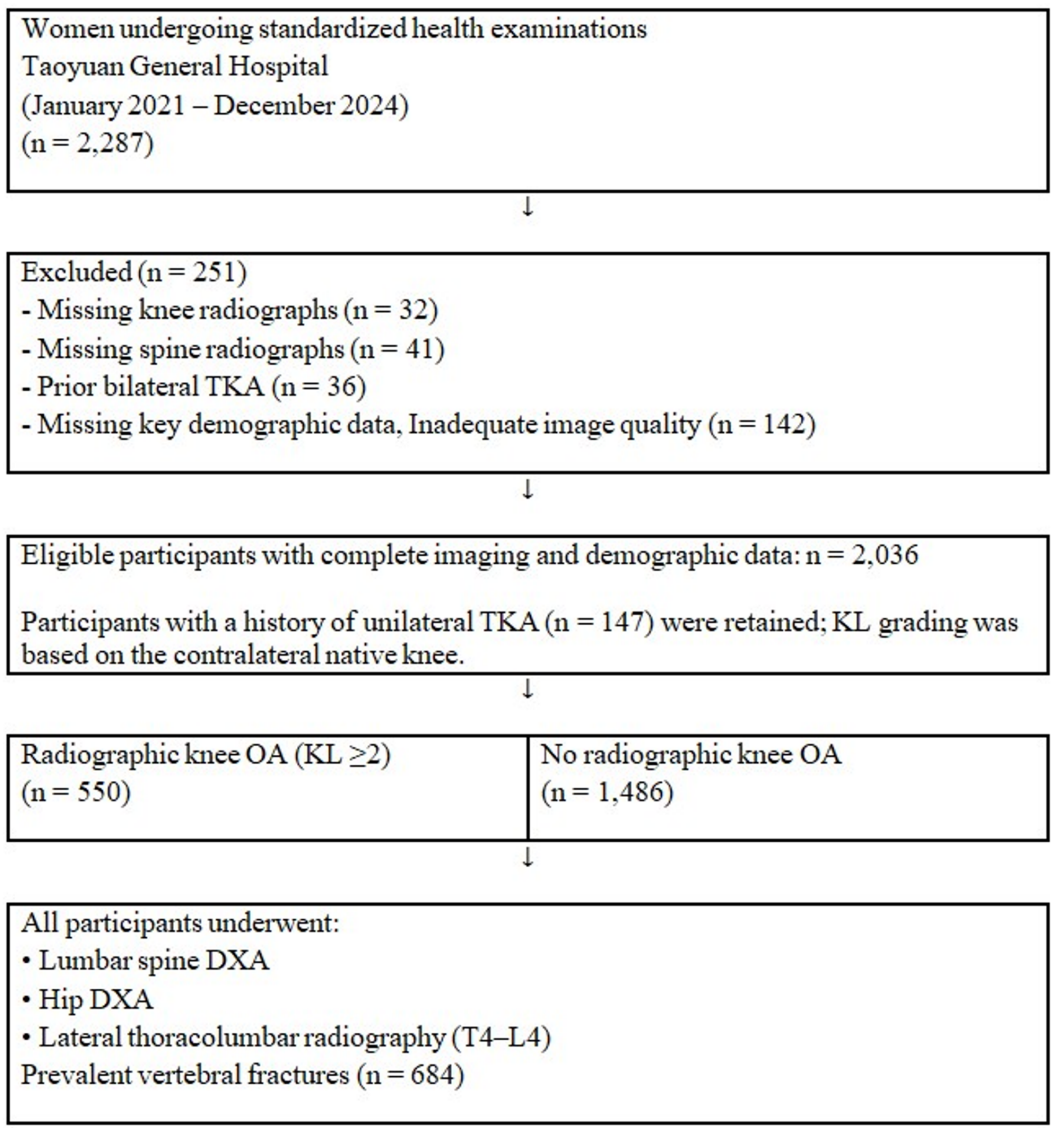
Flow diagram of participant selection. A total of 2,287 women underwent standardized health examinations between January 2021 and December 2024. After excluding 251 participants with incomplete imaging data, prior bilateral total knee arthroplasty, or inadequate image quality, 2,036 women were included in the final cross-sectional analysis

A flow diagram of participant selection is provided in Fig. 1.

### Anthropometric assessment

Age, height, and body weight were measured during the health examination. Body mass index (BMI) was calculated as weight (kg) divided by height squared (m²).

### Bone mineral density measurement

Bone mineral density (BMD) measurements of the bilateral hips and lumbar spine (L1–L4) were obtained using dual-energy X-ray absorptiometry (DXA) with the Lunar Prodigy system (General Electric, Fairfield, CT, USA), and results were expressed in g/cm2. T-scores were calculated using the NHANES III (National Health and Nutritional Examination Survey) reference database derived from healthy white women aged 20–40 years for both lumbar spine and hip measurements. In accordance with the World Health Organization (WHO) diagnostic criteria [20], osteoporosis was defined as a T-score ≤ − 2.5; low bone mass as a T-score between − 1.0 and − 2.5; and normal bone density as a T-score > − 1.0. Spine BMD was measured as the average of L1–L4. Vertebrae affected by compression fractures, vertebroplasty, metallic implants, or overlying calcifications were excluded from analysis in accordance with ISCD (International Society for Clinical Densitometry) guidelines [21]. Metallic implants or prostheses in the affected hip were also excluded when measuring the hip BMD according to a previous study [22]. T-scores were recorded for the lumbar spine (L1–L4), right hip, and left hip.

### Assessment of radiographic knee osteoarthritis

The conventional radiographs for this study were acquired using Toshiba Diagnostic X-Ray System (MRAD-A50S). Bilateral anteroposterior, lateral (30° flexion), and weight-bearing anteroposterior radiographs of the knees were obtained. Radiographic changes relating to OA were assessed using the Kellgren-Lawrence (KL) grading scale [23]. Images of the patellofemoral and femorotibial compartments were graded independently by two board-certified orthopaedic surgeons, and concordant grades were accepted. When there was a difference of one grade between the two readers, the higher grade was accepted. If the discrepancy exceeded one grade, a third board-certified orthopaedic surgeon was consulted, and the grade concordant with the third reviewer’s grade was accepted. The inter-rater agreement within one grade between the two readers was 94.2% [24, 25]. If there were findings suggestive of secondary causes of OA or joint replacement, the grading was based on findings in the intact knees. Participants who had undergone bilateral knee replacement and those with images of inappropriate quality for reading were excluded. Participants with unilateral TKA were not excluded, as knee osteoarthritis status was determined using radiographic Kellgren–Lawrence grading of the contralateral non-operated knee. A total of 147 women had a history of unilateral TKA. Participants with radiographic knee OA were defined as KL grade ≥ 2 in at least 1 knee.

### Vertebral fracture assessment

Lateral thoracolumbar spine radiographs spanning T4 to L4 were evaluated for vertebral compression fractures. Vertebral fractures were assessed using Genant’s semi-quantitative (SQ) grading method [26]. Vertebral deformities were classified as:

- Grade 0 (normal)
- Grade 1 (mild): 20–25% reduction in anterior, middle, or posterior vertebral height
- Grade 2 (moderate): 25–40% height reduction
- Grade 3 (severe): >40% height reduction

A prevalent vertebral fracture was defined as Genant Grade ≥ 1, corresponding to a reduction of at least 20% in vertebral height in any dimension.

To minimize misclassification in the presence of degenerative spinal changes, vertebral wedging due to osteoarthritis-related remodeling, endplate irregularities, and non-fracture deformities were differentiated from osteoporotic fractures based on vertebral shape, cortical disruption, and endplate contour. Cases with uncertainty were jointly reviewed and resolved by consensus. Two board-certified orthopaedic surgeons independently assessed vertebral fractures while blinded to knee OA status. Discrepancies were resolved by consensus discussion. Inter-rater reliability was assessed using Cohen’s kappa statistic.

### Statistical analysis

Continuous variables are presented as mean ± standard deviation (SD), and categorical variables as counts (percentages).

Baseline characteristics were compared using Welch’s t-test for continuous variables and chi-square tests for categorical variables.

### Association between knee oa and vertebral fracture

Logistic regression analyses were performed to evaluate the association between knee OA and prevalent vertebral fracture.

Sequential models were constructed:

- Model 0: Crude
- Model 1: Adjusted for age
- Model 2: Adjusted for age and BMI

Odds ratios (ORs) and 95% confidence intervals (CIs) were reported

### Factors associated with knee OA

To evaluate associations between knee OA and BMD, logistic regression models were constructed including:

- Age
- BMI
- Lumbar spine T-score
- Mean hip T-score.

To assess multicollinearity, Variance Inflation Factors (VIF) were calculated for all predictors. A VIF > 5 was considered indicative of significant collinearity.

Additional models were conducted separately including:

- Age + BMI + lumbar spine T-score
- Age + BMI + mean hip T-score

These analyses were performed to evaluate site-specific associations without collinearity influence.

### Stratified analyses

Stratified analyses were conducted by age group and BMI category to explore potential effect modification. All statistical tests were two-sided, and *p* < 0.05 was considered statistically significant. Analyses were performed using SPSS version 25.0 (IBM Corp., Armonk, NY, USA). To evaluate the potential impact of unmeasured confounding, we calculated the E-value for the fully adjusted odds ratio and its confidence interval. The E-value represents the minimum strength of association that an unmeasured confounder would need to have with both knee osteoarthritis and vertebral fracture prevalence, conditional on measured covariates, to fully explain away the observed association.

### Covariate availability

Information on diabetes mellitus, chronic kidney disease, glucocorticoid use, anti-osteoporotic medications, physical activity, and falls history was not available in the database and therefore could not be included in multivariable models. This limitation is addressed in the Discussion.

## Results

### Baseline characteristics

A total of 2,036 postmenopausal women were included in the analysis. The mean age was 73.5 ± 10.4 years, and the mean BMI was 24.2 ± 3.8 kg/m². Radiographic knee osteoarthritis (KL ≥ 2) was present in 550 participants (27.0%). The mean lumbar spine T-score was − 2.03 ± 1.49, while mean left and right hip T-scores were − 1.79 ± 1.17 and − 1.79 ± 1.20, respectively. Prevalent vertebral fractures were identified in 684 women (33.6%) (Table 1). A total of 147 participants had undergone unilateral TKA and were included in the analysis using the contralateral native knee for OA classification (Fig. 1).

**Table 1:** Baseline characteristics of the study population

Characteristic	*N*	Mean ± SD / *n* (%)
Age (years)	2036	73.46 ± 10.37
Height (cm)	2036	153.07 ± 6.28
Weight (kg)	2036	56.65 ± 9.60
BMI (kg/m²)	2036	24.17 ± 3.78
Lumbar spine T-score	2026	-2.03 ± 1.49
Left hip T-score	1951	-1.79 ± 1.17
Right hip T-score	1949	-1.79 ± 1.20
Knee osteoarthritis (KL ≥ 2), n (%)	2036	550 (27.0%)
Vertebral fracture, n (%)	2036	684 (33.6%)

Continuous variables are presented as mean ± SD. OA was defined as Kellgren–Lawrence (KL) grade ≥ 2 in at least one knee

*Abbreviations* *BMI* body mass index, *OA* osteoarthritis

### Comparison between participants with and without knee osteoarthritis

Women with radiographic knee OA were significantly older than those without OA (78.03 ± 8.30 vs. 71.77 ± 10.55 years, *p* < 0.00001) and had higher BMI (25.75 ± 3.86 vs. 23.58 ± 3.58 kg/m², *p* < 0.0001). Lumbar spine T-scores were significantly higher in women with OA compared with those without OA (− 1.84 ± 1.44 vs. − 2.09 ± 1.50, *p* = 0.00059). In contrast, mean hip T-scores did not differ significantly between groups. Prevalent vertebral fractures were more common among women with OA than among those without OA (41.5% vs. 30.7%, *p* < 0.0001) (Table 2).

**Table 2:** Comparison of characteristics between participants with and without knee osteoarthritis (OA)

Variable	No OA (*n* = 1486)	OA (*n* = 550)	*P* value
Age (years)	71.77 ± 10.55	78.03 ± 8.30	< 0.00001
Height (cm)	153.45 ± 6.32	152.03 ± 6.04	< 0.00001
Weight (kg)	55.57 ± 9.29	59.57 ± 9.80	< 0.00001
BMI (kg/m²)	23.58 ± 3.58	25.75 ± 3.86	< 0.00001
Lumbar spine T-score	-2.09 ± 1.50 (*n* = 1481)	-1.84 ± 1.44 (*n* = 545)	0.00059
Left hip T-score	-1.79 ± 1.18 (*n* = 1428)	-1.81 ± 1.13 (*n* = 523)	0.64547
Right hip T-score	-1.79 ± 1.23 (*n* = 1426)	-1.79 ± 1.11 (*n* = 523)	0.94891

Values are presented as mean ± SD. *P* values were calculated using Welch’s t-test for continuous variables. Sample sizes for BMD T-scores may be smaller due to exclusion of vertebrae or hips with artifacts or incomplete measurements

### Association between knee osteoarthritis and vertebral fracture prevalence

Among 684 women with prevalent vertebral fractures (Genant Grade ≥ 1), 336 (49.1%) were classified as Grade 1 (mild), 203 (29.7%) as Grade 2 (moderate), and 145 (21.2%) as Grade 3 (severe). Moderate-to-severe fractures (Grade ≥ 2) accounted for 347 cases (50.7%) of all vertebral fractures (Supplementary Table S1). Agreement between the two independent reviewers was high (κ = 0.82, 95% CI 0.78–0.86), corresponding to almost perfect agreement according to conventional benchmarks. All discrepant cases were adjudicated by consensus discussion. In crude analysis, radiographic knee OA was associated with higher odds of prevalent vertebral fracture (OR 1.60, 95% CI 1.31–1.96; *p* < 0.0001). After adjustment for age alone, the association was attenuated (OR 1.01, 95% CI 0.81–1.26). After further adjustment for both age and BMI, the association was no longer statistically significant (adjusted OR 0.95, 95% CI 0.76–1.20; *p* = 0.6800) (Table 3). In sensitivity analyses, the E-value for the fully adjusted odds ratio (0.95) was 1.16, and the E-value for the upper confidence limit was 1.00, indicating that only a weak unmeasured confounder would be required to shift the estimate to the null. Given that the observed association was already statistically non-significant, substantial residual confounding would be unlikely to alter the overall interpretation. The percentage reduction in the odds ratio from crude to fully adjusted models indicated substantial confounding by age and BMI. Stratified analyses by age group and BMI category are shown in Table 4. Vertebral fracture prevalence increased markedly with age, from 9.9% in women aged 50–64 years to 50.2% in those aged ≥ 75 years. However, knee OA was not significantly associated with vertebral fracture within any age or BMI subgroup. Likelihood ratio tests demonstrated no statistically significant interaction between knee OA and age group (P_interaction = 0.549) or BMI category (P_interaction = 0.688). These analyses were exploratory and may have been underpowered in certain strata.

**Table 3:** Association between radiographic knee osteoarthritis and prevalent vertebral fracture

Variable	Vertebral fracture *n*/*N* (%)	Model 0 Crude OR (95% CI)	*p* value	Model 1 Age-adjusted OR (95% CI)	*p* value	Model 2 Age + BMI-adjusted OR (95% CI)	*p* value
Non-OA	456 / 1,486 (30.7)	Reference	—	Reference	—	Reference	—
OA	228 / 550 (41.5)	1.60 (1.31–1.96)	< 0.001	1.01 (0.81–1.26)	0.913	0.95 (0.76–1.20)	0.680

Odds ratios were estimated using logistic regression. Model 0: crude; Model 1: adjusted for age; Model 2: adjusted for age and body mass index (BMI)

*Abbreviations: n/N, number of participants with vertebral fracture/total number of participants in each group;* *OA* osteoarthritis, *OR* odds ratio, *CI* confidence interval

**Table 4:** Stratified analysis of the association between knee osteoarthritis and prevalent vertebral fracture

Stratification	*N*	Fracture *n* (%)	OA OR (95% CI)	*p* value	P_interaction
Age group					0.549
50–64 years	414	41 (9.9%)	1.57 (0.56–4.41)	0.395	
65–74 years	707	184 (26.0%)	1.08 (0.72–1.63)	0.707	
≥ 75 years	915	459 (50.2%)	0.91 (0.66–1.26)	0.575	
BMI category					0.688
< 18.5 kg/m²	91	28 (30.8%)	1.22 (0.58–2.55)	0.604	
18.5–23.9 kg/m²	966	312 (32.3%)	1.04 (0.76–1.42)	0.804	
≥ 24 kg/m²	979	344 (35.1%)	0.89 (0.59–1.34)	0.572	

Odds ratios were estimated using logistic regression models within each stratum. Unless stratified by the respective variable, models were adjusted for age and body mass index (BMI)

P_interaction values were derived from likelihood ratio tests comparing models with and without multiplicative interaction terms

### Factors associated with radiographic knee osteoarthritis

In univariable logistic regression analyses, increasing age, higher BMI, higher body weight, and higher lumbar spine T-score were significantly associated with knee OA, whereas greater height was inversely associated. In multivariable models including age, BMI, lumbar spine T-score, and mean hip T-score, older age (OR 1.05 per year, 95% CI 1.04–1.06; *p* < 0.0001) and higher BMI (OR 1.12 per kg/m², 95% CI 1.09–1.15; *p* < 0.0001) remained independently associated with knee OA. Higher lumbar spine T-score was independently associated with knee OA (OR 1.28, 95% CI 1.17–1.40; *p* < 0.0001), whereas mean hip T-score was not independently associated (OR 0.97, 95% CI 0.88–1.06; *p* = 0.42) (Table 5).

**Table 5:** Multivariable model for factors associated with knee osteoarthritis

Variable	OR	95% CI	*p* value
Age (per year)	1.05	1.04–1.06	< 0.0001
BMI (kg/m²)	1.12	1.09–1.15	< 0.0001
Lumbar spine T-score	1.28	1.17–1.40	< 0.0001
Mean hip T-score	0.97	0.88–1.06	0.4200

Odds ratios were estimated using multivariable logistic regression. The model included age, body mass index (BMI), lumbar spine T-score, and mean hip T-score simultaneously

Variance Inflation Factors (VIFs) for all predictors were < 2.0, indicating no significant multicollinearity

*OA* osteoarthritis, *OR* odds ratio, *CI* confidence interval

### Variance Inflation Factors for all predictors were below 2.0, indicating no significant multicollinearity

In additional models examining skeletal sites separately:

- Lumbar spine T-score remained significantly associated with knee OA after adjustment for age and BMI
- Mean hip T-score remained non-significant

### Summary of site-specific findings

Radiographic knee OA was associated with higher lumbar spine BMD but not with hip BMD after adjustment for age and BMI. Although vertebral fractures were more prevalent among women with OA in unadjusted comparisons, this association was fully explained by differences in age and BMI.

## Discussion

In this cross-sectional study of older Asian women, radiographic knee osteoarthritis (OA) was associated with higher lumbar spine bone mineral density (BMD) but not with hip BMD after adjustment for age and body mass index (BMI). Although women with knee OA exhibited a higher crude prevalence of vertebral fractures, this association was fully attenuated after accounting for age and BMI. These findings suggest that the apparent excess of vertebral fractures in women with OA largely reflects differences in aging and adiposity rather than indicating an independent relationship between OA and vertebral fracture prevalence.

### Site-specific dissociation between lumbar and hip BMD

The observation that lumbar spine BMD was higher in women with knee OA is consistent with prior epidemiologic studies reporting elevated axial BMD in individuals with radiographic OA [6–9]. However, DXA-derived lumbar spine measurements are particularly susceptible to degenerative artifacts. Osteophyte formation, facet joint hypertrophy, endplate sclerosis, and vascular calcifications can increase areal BMD independent of trabecular bone mass [10, 11]. Thus, elevated spine BMD in OA populations may not represent improved skeletal strength.

In contrast, hip BMD—less affected by degenerative artifact—was not independently associated with knee OA after adjustment. Several explanations may account for this dissociation. Increased body weight may simultaneously increase mechanical loading and OA prevalence; once BMI is controlled for, the independent association weakens. Furthermore, knee pain and reduced mobility may alter weight-bearing patterns and physical activity, potentially attenuating loading-related increases in proximal femoral BMD. Prior studies have similarly reported inconsistent or null associations between knee OA and hip BMD after accounting for confounders [12, 16–18]. Together, these findings support a site-specific interpretation of the OA–BMD relationship.

#### Vertebral fracture prevalence and confounding

The observed vertebral fracture prevalence of 33.6% in our cohort may initially appear higher than estimates reported in general adult populations. However, this finding is consistent with age-specific data from Asian populations. Age-stratified analyses in postmenopausal Chinese women demonstrated a marked increase in vertebral fracture prevalence from 13% in women aged 50–59 years to over 50% in those aged ≥ 80 years [27]. Given that the mean age of our cohort was 73.5 years, a prevalence in the range of 30–40% aligns with these age-specific estimates. Furthermore, data from aging Hakka women in Taiwan demonstrated that fragility fractures were present in 44% of participants, with vertebral fractures being the most common fracture type [28]. These findings suggest that vertebral fractures are highly prevalent in older East Asian women, particularly in super-aged or ethnically distinct populations. Importantly, our study utilized systematic lateral thoracolumbar radiography (T4–L4) as part of standardized health screening, which increases detection of morphometric vertebral fractures compared with symptom-based ascertainment. Therefore, the observed prevalence likely reflects the advanced age structure of the cohort and comprehensive radiographic assessment rather than referral bias. Nevertheless, our screening population may not fully represent younger community-dwelling women, and generalizability should be interpreted accordingly.

Although crude analyses demonstrated a higher prevalence of vertebral fractures among women with OA, the association was no longer observed after adjustment for age and BMI. The marked attenuation of the odds ratio indicates that age and adiposity explain most of the observed difference. Knee OA is strongly age-dependent and more prevalent among individuals with higher BMI, both of which are independently associated with vertebral fracture prevalence.

These findings suggest that knee OA may function primarily as a marker of aging and body composition rather than as an independent determinant of vertebral fracture prevalence. Importantly, the absence of an independent association in adjusted models does not imply that OA is protective or harmful with respect to fractures; rather, it indicates that shared risk factors likely account for the crude association.

### Bone quantity versus bone quality

DXA-derived BMD reflects bone quantity but does not directly assess bone microarchitecture or material properties. In OA populations, elevated spine BMD may coexist with impaired bone quality. Measures such as trabecular bone score (TBS), bone turnover markers, or high-resolution peripheral quantitative computed tomography (HR-pQCT) may provide complementary information regarding trabecular integrity and cortical porosity. Prior studies have suggested that individuals with OA may exhibit alterations in bone remodeling that are not fully captured by areal BMD alone [14–16]. Therefore, elevated lumbar spine BMD in OA should not be interpreted as evidence of preserved bone strength without consideration of bone quality parameters.

#### Degenerative artifacts and clinical interpretation

Degenerative changes in the lumbar spine are common in older adults and may substantially influence DXA interpretation. According to ISCD recommendations [21], affected vertebrae should be excluded when possible, and clinicians should interpret lumbar spine BMD cautiously in individuals with significant degenerative disease. In patients with knee OA—who frequently exhibit concomitant spinal degeneration—lumbar spine BMD may overestimate true trabecular density. In such cases, greater emphasis on hip BMD and comprehensive clinical fracture history may provide a more reliable assessment of skeletal fragility.

#### Alternative explanations and shared aging pathways

The relationship between OA and vertebral fractures is likely multifactorial. In addition to degenerative artifacts and confounding by age and BMI, shared aging-related mechanisms may contribute. Sarcopenia, reduced mobility, chronic low-grade inflammation, and metabolic dysfunction may simultaneously influence joint degeneration and skeletal health. Conversely, vertebral fractures may alter posture and biomechanics, potentially affecting knee loading patterns. Given the cross-sectional design, the temporal sequence between OA onset and fracture occurrence cannot be determined, and bidirectional pathways cannot be excluded.

#### Strengths and limitations

Residual confounding cannot be entirely excluded despite adjustment for clinically relevant covariates. To quantify the potential impact of unmeasured confounding, we conducted an E-value sensitivity analysis. The E-value for the fully adjusted odds ratio (0.95) was 1.16, and the E-value for the upper confidence limit was 1.00. These results indicate that the observed association was close to the null even before consideration of unmeasured confounding. Because the association was already statistically non-significant after age adjustment, residual confounding would be unlikely to materially change the substantive interpretation of the findings.

In particular, diabetes mellitus has been associated with preserved or elevated BMD but increased fracture risk due to impaired bone quality, a phenomenon often described as the “diabetes paradox.” Although diabetes status was unavailable in this dataset, the absence of an independent association between knee OA and vertebral fracture after age adjustment suggests that such metabolic factors are unlikely to fully account for the observed findings.

This study has several strengths, including its large sample size, standardized radiographic assessment of knee OA, use of Genant semi-quantitative grading for vertebral fractures, and simultaneous evaluation of site-specific BMD and vertebral fracture prevalence within a unified analytic framework.

Several limitations should be considered. First, the cross-sectional design precludes inference regarding temporal sequence or causality. Second, information on diabetes, medication use, falls history, and physical activity was unavailable and could not be incorporated into multivariable models. Third, vertebral fractures were assessed using supine lateral thoracolumbar radiography (T4–L4), which may underestimate fracture prevalence compared with standing films. Although inter-rater reliability was high (κ = 0.82), intra-rater reproducibility was not formally evaluated and may introduce additional measurement variability. Fourth, radiographic OA does not necessarily reflect symptomatic burden, and functional status measures were not available. Fifth, T-scores were calculated using the NHANES III reference database derived from healthy white women aged 20–40 years, which is the standard reference embedded in the GE Lunar Prodigy system and widely used in clinical practice according to WHO diagnostic criteria. Although ethnic differences in normative BMD distributions may exist, the use of this reference facilitates comparability with prior epidemiological studies and international diagnostic thresholds. Finally, the single-center design may limit generalizability to other populations or healthcare settings.

### Clinical implications

From a clinical perspective, these findings underscore the need for cautious interpretation of lumbar spine BMD in women with radiographic knee OA. Elevated spine T-scores in this population may reflect degenerative artifact rather than enhanced skeletal strength. Hip BMD and documented fracture history may provide more robust indicators of skeletal fragility. Clinicians should avoid assuming that higher lumbar spine BMD in patients with OA confers protection against vertebral fractures.

## Supplementary Information

Supplementary Material 1.

## Data Availability

The datasets generated and/or analysed during the current study are not publicly available due to restrictions related to patient privacy and institutional regulations but are available from the corresponding author on reasonable request and with permission of Taoyuan General Hospital.
