# Supplementary material for: Radiographic knee osteoarthritis is associated with higher lumbar spine bone mineral density but not reduced vertebral fracture prevalence: a retrospective cross-sectional study in older Asian women
Source: BMC Musculoskelet Disord. 2026 Mar 30;27:403. doi: 10.1186/s12891-026-09751-8 (PMC13154486; doi:10.1186/s12891-026-09751-8)
Supplement: Supplementary file 1 — Supplementary Material 1. [file 12891_2026_9751_MOESM1_ESM.docx]

**Supplementary Table S1. Distribution of vertebral fractures according to Genant semi-quantitative grading**

| **Genant Grade** | **Definition** | **Number (n)** | **Percentage (%)** |
| --- | --- | --- | --- |
| Grade 1 | 20–25% vertebral height reduction | 336 | 49.1 |
| Grade 2 | 25–40% vertebral height reduction | 203 | 29.7 |
| Grade 3 | >40% vertebral height reduction | 145 | 21.2 |
| Total (Grade ≥1) | — | 684 | 100 |

Vertebral fractures were assessed on lateral thoracolumbar radiographs (T4–L4) using Genant’s semi-quantitative method. Grade ≥1 was defined as ≥20% vertebral height reduction.
